# Exploring the genomic and transcriptomic profiles of glycemic traits and drug repurposing

**DOI:** 10.1186/s12929-025-01137-7

**Published:** 2025-05-21

**Authors:** Min-Rou Lin, Cheng-Lin Tsai, Cai-Sian Liao, Chun-Yu Wei, Wan-Hsuan Chou, Tzu-Hung Hsiao, Wei-Chiao Chang

**Affiliations:** 1 Department of Clinical Pharmacy, School of Pharmacy, Taipei Medical University, Taipei, 110 Taiwan; 2 Bioinformatics Program, Institute of Statistical Science, Taiwan International Graduate Program, Academia Sinica, Taipei, 110 Taiwan; 3 Bioinformatics Program, Taiwan International Graduate Program, National Taiwan University, Taipei, 110 Taiwan; 4 Department of Medical Research, Taichung Veterans General Hospital, 1650 Taiwan Boulevard Sect. 4, Taichung, 407219 Taiwan; 5 Department of Public Health, Fu Jen Catholic University, New Taipei City, 242 Taiwan; 6 Institute of Genomics and Bioinformatics, National Chung Hsing University, Taichung, 402 Taiwan; 7 Master Program in Clinical Genomics and Proteomics, School of Pharmacy, Taipei Medical University, Taipei, 110 Taiwan; 8 Core Laboratory of Neoantigen Analysis for Personalized Cancer Vaccine, Office of R&D, Taipei Medical University, Taipei, 110 Taiwan; 9 Integrative Research Center in Critical Care, Wan Fang Hospital, Taipei Medical University, Taipei, 116 Taiwan; 10 Department of Pharmacy, Wan Fang Hospital, Taipei Medical University, Taipei, 116 Taiwan; 11 Department of Pharmacology, National Defense Medical Center, Taipei, 114 Taiwan

**Keywords:** Drug repurposing, Fasting glucose, Genome-wide association study, Glycemic traits, Hemoglobin A1c, Polygenic risk score, Transcriptome-wide association study, Type 2 diabetes

## Abstract

**Background:**

Type 2 diabetes is an increasingly prevalent metabolic disorder with moderate to high heritability. Glycemic indices are crucial for diagnosing and monitoring the disease. Previous genome-wide association study (GWAS) have identified several risk loci associated with type 2 diabetes, but data from the Taiwanese population remain relatively sparse and primarily focus on type 2 diabetes status rather than glycemic trait levels.

**Methods:**

We conducted a comprehensive genome-wide meta-analysis to explore the genetics of glycemic traits. The study incorporated a community-based cohort of 145,468 individuals and a hospital-based cohort of 35,395 individuals. The study integrated genetics, transcriptomics, biological pathway analyses, polygenic risk score calculation, and drug repurposing for type 2 diabetes.

**Results:**

This study assessed hemoglobin A1c and fasting glucose levels, validating known loci (*FN3K*, *SPC25*, *MTNR1B*, and *FOXA2*) and discovering new genes, including *MAEA* and *PRC1*. Additionally, we found that diabetes, blood lipids, and liver- and kidney-related traits share genetic foundations with glycemic traits. A higher PRS was associated with an increased risk of type 2 diabetes. Finally, eight repurposed drugs were identified with evidence to regulate blood glucose levels, offering new avenues for the management and treatment of type 2 diabetes.

**Conclusions:**

This research illuminates the unique genetic landscape of glucose regulation in Taiwanese Han population, providing valuable insights to guide future treatment strategies for type 2 diabetes.

**Supplementary Information:**

The online version contains supplementary material available at 10.1186/s12929-025-01137-7.

## Background

The prevalence of type 2 diabetes among the Taiwanese population has significantly increased. From 2005 to 2014, Taiwan experienced a 66% increase in type 2 diabetes cases [1], with the condition’s prevalence reaching an estimated 10.9% by 2017 [2]. Type 2 diabetes is characterized by the body’s inability to regulate glucose levels properly, either due to insufficient insulin production by the pancreas or because cells develop insulin resistance. Over time, the accumulation of glucose in the bloodstream can lead to serious health complications, including renal and cardiovascular diseases. The cornerstone of diagnosing and managing type 2 diabetes lies in monitoring glycemic control, primarily through measuring blood sugar levels. Key indicators include hemoglobin A1c (HbA1c) and fasting glucose (FG) levels, which respectively offer insights into long-term and short-term glucose control. Elevated levels of these biomarkers are indicative of poor glycemic control, a major risk factor for the onset and progression of type 2 diabetes and its associated complications.

Over the past decades, numerous genetic loci have been linked to an increased risk of type 2 diabetes in Asian populations, enriching our understanding of the disease's complex genetic foundation. In the Taiwanese population, several studies have identified loci associated with glycemic phenotypes and type 2 diabetes susceptibility, including *HACL1*, *RAD21*, *ASH1L*, *GAF*, *KCNQ1*, *PTPRD*, and *SRR* [3–5]. Similarly, a large meta-analysis of GWAS in Japanese ancestry revealed 88 loci significantly associated with type 2 diabetes, including novel variants with distinct minor allele frequencies (MAF) compared to European populations, such as those located on *GP2* and *GLP1R* [6]. GWAS of Southern Han Chinese descent have identified rs10229583 near *PAX4* as a novel locus for type 2 diabetes [7]. Moreover, a three-stage GWAS conducted in the Han Chinese population replicated several known loci and identified novel susceptibility loci, inclusing *GRK5* and *RASGRP1* [8]. Although existing studies in Asian populations have provided valuable insights into the genetic architecture of type 2 diabetes, many of these investigations, particularly those focusing on the Taiwanese population, are limited by relatively small sample sizes compared to studies conducted on Europeans. This underscores the need for a more comprehensive, large-scale multi-omics study to better characterize the genetic and molecular mechanisms of type 2 diabetes within the Taiwanese Han population.

To bridge this gap and enhance the genetic insights into type 2 diabetes, we conducted a examination of genomic and transcriptomic data related to glycemic traits. By integrating diverse methodologies, we extend beyond traditional GWAS to investigate underlying biological mechanisms through transcriptome-wide association study (TWAS) and to derive clinically actionable insights via drug repurposing. We also developed a polygenic risk score (PRS) to estimate an individual’s genetic predisposition to type 2 diabetes, providing a new way for early detection and risk stratification. Our study aims are to leverage expanded datasets and integrative bioinformatics approaches to advance understanding of the genetic architecture and to explore alternative treatment options for type 2 diabetes in the Taiwanese population.

## Methods

### Study designs

**Fig. 1:**
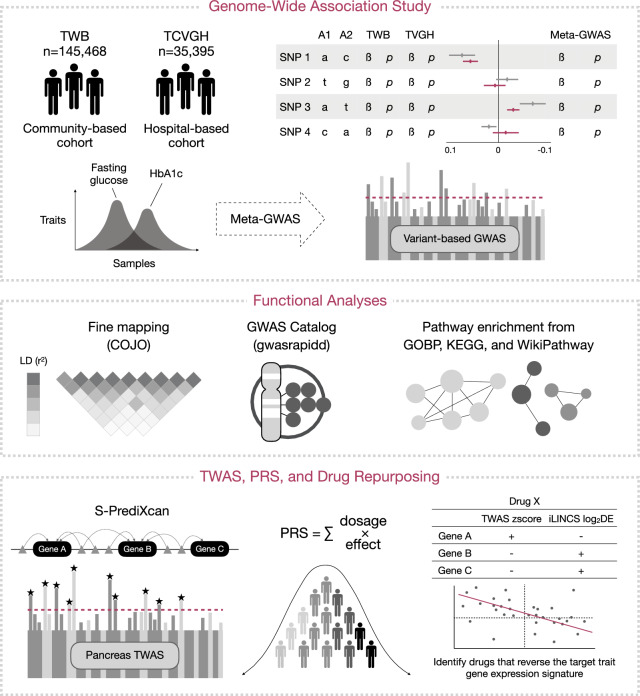
Study Workflow. The study conducted GWAS for fasting glucose and HbA1c using a community-based cohort from TWB and a hospital-based cohort from TCVGH, followed by a meta-analysis of the summary statistics from both cohorts. Fine mapping was performed to identify independent signals from the GWAS. The GWAS catalog was queried to distinguish known and novel variants. Pathway enrichment analysis was carried out to investigate the biological functions of the identified risk genes. Additionally, TWAS was conducted to explore risk genetic factors at the gene expression level. PRS were calculated for both glycemic traits to predict the risk of developing type 2 diabetes. Finally, drug repurposing strategies were applied to identify potential treatments for type 2 diabetes

This study utilized two Taiwanese cohorts: a community-based cohort from the TWB and a hospital-based cohort from Taichung Veterans General Hospital (TCVGH), with a combined sample size of 180,863 individuals. GWAS analyses were independently conducted on the two cohorts, followed by a meta-analysis to integrate their summary statistics. To gain deeper insights into the biological significance of the identified genetic loci, functional analyses were performed, including fine mapping, gene annotation, and pathway enrichment analyses. Additionally, a TWAS was conducted using imputed gene expression data derived from the expression quantitative loci (eQTL) data of pancreas tissue. Furthermore, PRS were calculated based on the glycemic trait GWAS, and their associations with various human diseases were evaluated using TWB data. Lastly, drug repurposing analyses were carried out by comparing drug activity-related gene expression profiles with glycemic trait-associated gene expression profiles to identify potential therapeutic candidates for type 2 diabetes. The overall study workflow is illustrated in Figure 1.

### Study population and genotyping

For the TWB cohort, genomic DNA was extracted from peripheral blood using Chemagen Blood DNA isolation kit or QIAsymphony DNA Maxi Kit [9]. Genotyping was conducted using the TWB1 and TWB2 array, and the variants showing a missing rate > 2%, a MAF < 5%, and a Hardy-Weinberg equilibrium (HWE) p-value > 1 × 10^–6^ were excluded before imputation. Imputation was conducted via IMPUTE2 software based on the 1451 whole genome sequence data from TWB and the 504 East Asian panel from the 1000 Genome Project [10]. After imputation, variants with a missing rate > 5%, MAF < 1%, and an info score < 0.3 were further excluded. Individuals with a missing rate > 2%, a heterozygosity rate beyond the mean ± 3 SD, 3rd-degree relatives, and those not of East Asian descent were removed for further analysis. Following these data filtering steps, the final TWB cohort consisted of 145,468 individuals and 9,814,944 variants for subsequent analysis. This study was approved by the Taipei Medical University Joint Institutional Review Board (TMU-JIRB N201906005).

For the TCVGH cohort, human genomic DNA was extracted from peripheral blood leukocytes according to a standard experimental protocol [11]. Genotyping was performed using the TWB2 array, and samples with a call rate < 95% were removed. For SNP quality control, we excluded non-autosomal SNPs as well as SNPs with a missing call rate greater than 5%, an MAF < 1%, or those that significantly deviated from HWE (p-value < 1 × 10^–5^). Imputation was conducted via Minimac4 on the Michigan Imputation Server, using the East Asian reference panel from the 1000 Genome Project. The final TCVGH cohort consisted of 35,395 individuals and 2,048,729 variants for subsequent analysis. This study was approved by the Taichung Veterans General Hospital Joint Institutional Review Board (CE24204A).

### Genome-wide association analyses (GWAS)

For the TWB cohort, HbA1c and FG levels were measured once through blood tests. Values that reached the minimum detectable limit were recorded as “< 3.8” for HbA1c. To ensure consistency in the analysis, we converted these values to 3.8. For the TCVGH cohort, the biochemical data were retrieved from the hospital information system from 2000 to 2022. Outliers for FG were excluded using a cutoff of mean ± 3SD. An average value of both glycemic traits was calculated for subjects with multiple records. GWAS was carried out for each cohort using PLINK v2 [12]. To account for population structure, principal component analysis (PCA) was performed separately for each cohort. Prior to PCA, linkage disequilibrium pruning was carried out in PLINK v2 to exclude highly correlated SNPs, ensuring only independent variants were retained. The genetic relationship matrix (GRM) was then calculated using GCTA, and PCA was applied to the GRM to generate eigenvectors and eigenvalues. Both glycemic traits underwent inverse normal transformation prior to analysis. Linear regression under an additive model was performed, adjusting for sex, age, age^2^, and the top ten principal components. Moreover, summary statistics from both cohorts were used for meta-analysis. A fixed effects model was applied with an assumption of a shared true effect between the two Taiwanese populations for a certain genetic variant. The effect sizes were estimated and weighted by standard errors using METAL [13]. The results from meta-GWAS form the central focus of the study. Signals with p-value < 5 × 10^–8^ were considered significant and signals with p-value < 1 × 10^–5^ were suggestive. The results were visualized using the R package ‘ggplot2’.

### Conditional analysis, functional analysis, and pathway enrichment analysis

Conditional analysis was conducted using the GCTA COJO [14] method with default parameters to identify independent genetic loci. The linkage disequilibrium (LD) reference was constructed from individual-level imputed genotype data from TWB. To identify known risk loci associated with glycemic traits, the GWAS catalog was accessed through the R package ‘gwasrapidd’ (access date on August 21, 2024). Specifically, variants previously reported to be linked with HbA1c measurement (EFO_0004541) or fasting blood glucose measurement (EFO_0004465) were defined as known variants. Gene annotation was performed using the R package ‘biomaRt’. For pathway enrichment analysis, the ‘ClusterProfiler’ R package was employed, drawing from databases such as the Gene Ontology biological process (GOBP), the Kyoto Encyclopedia of Genes and Genomes (KEGG), and WikiPathways.

### Transcriptome-wide association analyses

For the TWAS of both glycemic traits, S-PrediXcan [15] was utilized to impute gene expression signatures from the summary statistics of the meta-GWAS. The imputation was based on the pancreas eQTL profile available in PredictDB. The choice of the pancreas tissue was guided by its biological relevance to glycemic traits, as both FG and HbA1c are closely linked to insulin secretion. Given the pancreas’s critical role in glucose regulation through insulin and glucagon secretion, it was considered the most appropriated tissue for this analysis. The significance of genes was determined after applying Bonferroni correction for multiple testing. The results were visualized using the R package ‘ggplot2’.

### Calculation of polygenic risk scores and drug repurposing

PRS was calculated as the sum of the allele doses weighted by the effect size. Summary statistics for both glycemic traits from the meta-GWAS were utilized to estimate the weights. Only significant variants were included for calculation. We conducted an association analysis between the glycemic trait-related PRS and various phenotypes using the ‘PheWAS’ R package. This analysis included 73 binary and 56 quantitative phenotypes sourced from the TWB. The binary phenotypes were derived from comprehensive questionnaires, while the quantitative phenotypes were acquired through blood or urine tests. We performed regression analyses that were adjusted for sex, age, age^2^, and the top ten principal components. To explore for alternative treatment options for type 2 diabetes, gene expression profiles of glycemic traits derived from the TWAS analysis were used for drug repurposing. Initially, genes with an FDR < 0.05 were selected. The genes with top and bottom 50 zscores were extracted as the gene expression signature for the glycemic traits. These glycemic trait gene expression signatures was then correlated with the drug perturbation gene expression signature from the iLINCS database [16]. Drugs with perturbation gene expression signatures that exhibit significant negative correlation with both the glycemic trait gene expression signatures (FDR < 0.05) were regarded as promising drug candidates to reverse the glycemic disease status. Additionally, a systematic literature search was conducted to identify studies relevant to these candidate drugs. Searches were performed in PubMed using a combination of drug names and keywords such as “glucose”, “glycemic”, “HbA1c”, and “kidney disease”. The search was restricted to studies published in English that provide preclinical or clinical evidence supporting the therapeutic effects of these drugs on serum glucose regulation.

## Results

### Genetic risk loci for HbA1c and FG

The first cohort consisted of 145,468 participants from the TWB, while the second cohort, the TCVGH cohort, included 35,359 participants. Both HbA1c and FG levels were examined in our analysis. Detailed baseline characteristics of the study population can be found in Table 1. The TWB cohort consists of subjects generally considered healthier, whereas the TCVGH cohort comprises hospital patients. Consequently, the clinical profiles differ significantly between the two cohorts, with all glycemic traits being slightly higher in the TCVGH cohort. The TCVGH cohort demonstrates a higher average age and a larger proportion of diabetes cases, along with a heightened prevalence of comorbidities such as hypertension, hyperlipidemia, and coronary artery disease.

**Table 1:** Baseline characteristics of the study cohorts

	TWB	TCVGH
Cohort size	145,468	35,359
Age (years, mean ± s.d.)	49.42 ± 11.38	60.78 ± 14.64
Sex (male, %)	36.24%	48.19%
BMI (mean ± s.d.)	24.27 ± 3.85	25.02 ± 3.97
HbA1c (%, mean ± s.d.)	5.76 ± 0.79	6.21 ± 1.06
Fasting glucose (mg/dL, mean ± s.d.)	96 ± 20.51	106.65 ± 21.11
Diabetes (%)	5.21^a^	32.39^b^
Hypertension (%)	12.07^a^	37.4^b^
Hyperlipidemia (%)	7.47^a^	40.34^b^
Coronary artery disease (%)	1.3^a^	20.32^b^

^a^Data obtained from the questionnaire

^b^Data obtained from ICD codes

The HbA1c GWAS in TWB revealed 4,055 significant genetic variants, with chromosome 17 exhibiting the strongest signal (Additional file 1: Figure S1, top panel, Additional file 2: Table S1). Similarly, chromosome 17 exist as the strongest signal after adjusted for the additional confounding factors, including BMI, eGFR, SGOT, SGPT, hemoglobin, and type 2 diabetes (Additional file 1: Figure S2). The HbA1c GWAS in TCVGH showed a similar pattern by identifying 598 significant variants (Additional file 1: Figure S1, bottom panel, Additional file 2: Table S2).

In the case of FG levels, the TWB GWAS identified 4,678 significant genetic variants, with chromosomes 2, 6, 7, 9, 11, 20 showing strong clustering (Additional file 1: Figure S3, top panel, Additional file 2: Table S3). The signals remain similar after adjusting for the additional confounding factors (Additional file 1: Figure S4). Meanwhile, the TCVGH GWAS identified 366 significant variants with the top signal located on chromosome 11 (Additional file 1: Figure S2, bottom panel, Additional file 2: Table S4).

### Meta-GWAS analysis between TWB and TCVGH cohorts

**Fig. 2:**
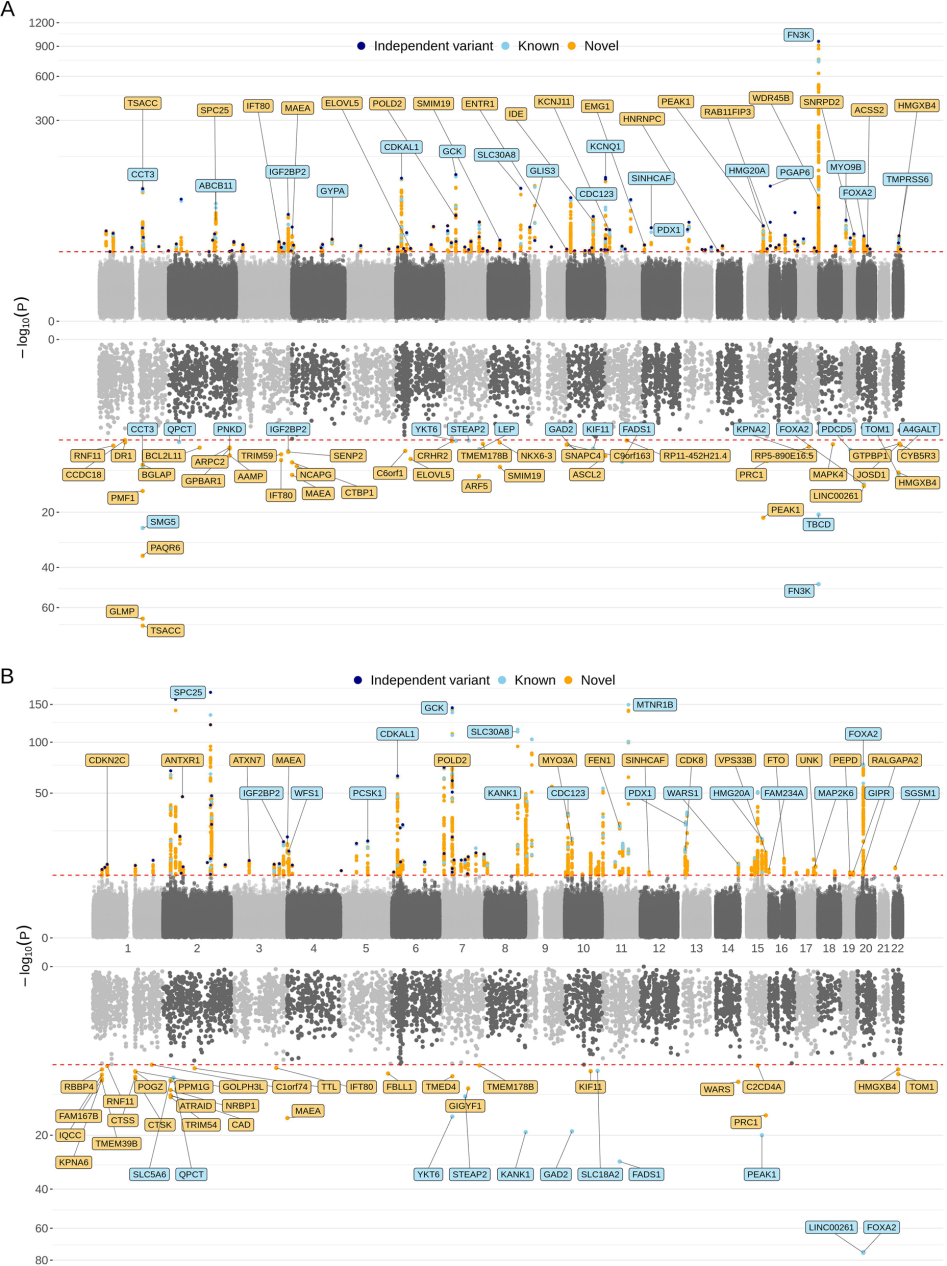
Miami plot of meta-GWAS and TWAS on HbA1c and fasting glucose. **A** Miami plot for HbA1c, with the upper panel showing meta-GWAS (TWB and TCVGH) and the lower panel showing TWAS results. **B** Miami plot for fasting glucose, with the upper panel showing meta-GWAS (TWB and TCVGH) and the lower panel showing TWAS results. Red horizontal dashed lines indicate significance thresholds (−log10(5 × 10^–8^) for meta-GWAS, −log10(6.89 × 10^–6^) for TWAS). Known loci are highlighted in blue, new associations from this study are in orange, and independent variants are in navy. We labeled only the top known and novel genes for each chromosome in the meta-GWAS

**Fig. 3:**
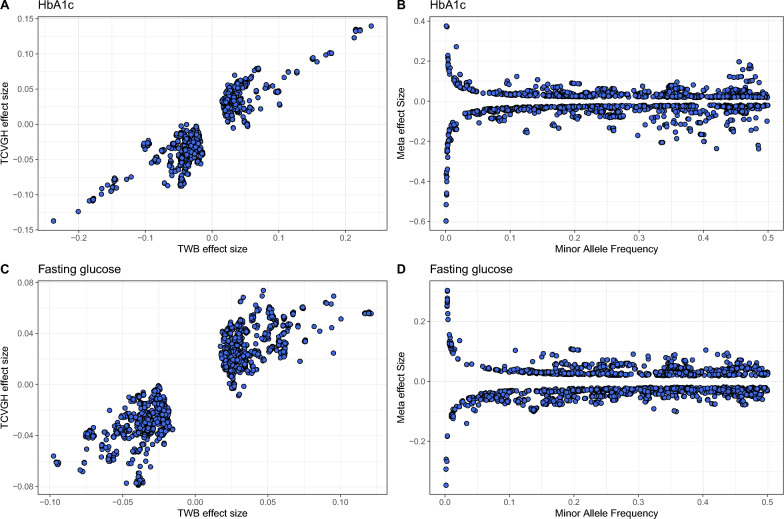
Scatter plot of effect sizes and minor allele frequencies (MAF). **A** The effect sizes in TWB (x-axis) and TCVGH (y-axis) of the 3600 overlapping significant variants in the HbA1c meta-GWAS. **B** The MAF (x-axis) and effect size (y-axis) of the 5394 significant variants in the HbA1c meta-GWAS. **C** The effect sizes in TWB (x-axis) and TCVGH (y-axis) of the 3298 overlapping significant variants in the fasting glucose meta-GWAS. **D** The MAF (x-axis) and effect size (y-axis) of the 5332 significant variants in the fasting glucose meta-GWAS

Meta-GWAS analysis of HbA1c revealed 5,394 significant variants in 186 genes (Figure 2A, top panel, Additional file 2: Table S5). The p-values of meta-GWAS analysis resulted in a genomic inflation factor (lambda) of 1.17. We also observed that variants with relatively smaller MAF tend to have larger effect sizes compared to variants with larger MAF (Figure 3B, 3D). Upon consulting the GWAS catalog, we identified 1,089 known variants within 696 genes, previously associated with HbA1c measurement (Additional file 2: Table S6). Among the known variants, 138 variants in 46 genes were successfully replicated in the current study (Additional file 2: Table S5). The top signal, rs113373052 in *FN3K*, has the same effect direction in both cohorts. Further conditional analysis identified 125 independent variants in 72 genes, including 47 known genes and 25 novel genes (Additional file 2: Table S7). Furthermore, we conducted a rigorous validation across the two cohorts, identifying 3,588 variants with a consistent and significant effect on HbA1c in both the community and hospital cohorts (Additional file 2: Table S5). The meta-analysis integrating these distinct cohorts provided a more robust and comprehensive assessment. Notably, among these 3,588 variants, 3,303 did not reach genome-wide significance in either of the individual cohorts but became significant after the meta-analysis. This underscores the enhanced statistical power achieved through a larger sample size, facilitating the detection of previously undetected signals.

In the Meta-GWAS analysis of FG, we identified 5,332 significant variants in 148 genes (Figure 2B, top panel, Additional file 2: Table S8). We further identified 60 independent variants in 23 genes, including 13 known genes and 10 novel genes (Additional file 2: Table S9). The p-values of meta-GWAS analysis resulted in a genomic inflation factor (lambda) of 1.16. A query of the GWAS catalog unveiled 430 known variants within 362 genes (Additional file 2: Table S10). Our Meta-GWAS successfully replicated 120 of these known variants, which are distributed across 36 genes (Additional file 2: Table S8). The top signal, rs1402837, was identified in the *SPC25* gene. It was exclusively detected in the TWB cohort and was calculated as an independent signal after conditional analysis. Validation across the two cohorts identify 3,276 variants with a consistent and significant effect on FG (Additional file 2: Table S8).

The study revealed 2148 shared variants between HbA1c and FG levels, including 68 genes as detailed in Additional file 2: Table S11. These shared variants consistently aligned in their effect direction across both glycemic traits, indicative of shared genetic backgrounds. Among the shared genes, 29 were known to be associated with both traits, while 25 were novel to both.

### TWAS predicts novel genes based on pancreas tissue

In the TWAS, 56 genes showed significant association of the pancreatic expression with HbA1c (Figure 2A, bottom panel), with 18 previously reported and 38 newly discovered in this study (Additional file 2: Table S12). Notably, *FN3K* was the most significant among the known genes (p-value = 1.74 × 10^–48^), aligning with the GWAS findings where 29 significant variants were found in the *FN3K* gene. Among the novel genes, *TSACC* showed the highest significance in the TWAS (p-value = 2.23 × 10^–71^), with its expression predicted by eQTL rs11264475. Another novel gene, *A4GALT* (p-value = 1.46 × 10^–7^), was predicted by three eQTLs (rs66781836, rs28910285, and rs5758951), all cis-eQTL within the 22q13.2 loci, marking it as the gene with predictions based on the most eQTLs among all significant genes.

Similarly, the TWAS for FG revealed 40 significant genes (Figure 2B, bottom panel), comprising 11 known and 29 newly identified genes (Additional file 2: Table S13). *FOXA2* emerged as the most significant known gene (p-value = 2.03 × 10^–75^), with predictions based on rs2277764. Among the novel genes, *MAEA* had the highest significance (p-value = 5.05 × 10^–16^), predicted by rs17164589, with 107 significant signals identified in this gene in the meta-GWAS.

In addition, we identified 16 shared genes between HbA1c and FG with consistent effect direction. Six of these genes were previously associated with both traits: *QPCT*, *YKT6*, *STEAP2*, *GAD2*, *FADS1*, and *FOXA2*, while six were novel to both: *RNF11*, *IFT80*, *MAEA*, *TMEM178B*, *PRC1*, and *HMGXB4*.

### Pathway analysis highlights insulin secretion as a key mechanism underlying glycemic trait-related genes

**Fig. 4:**
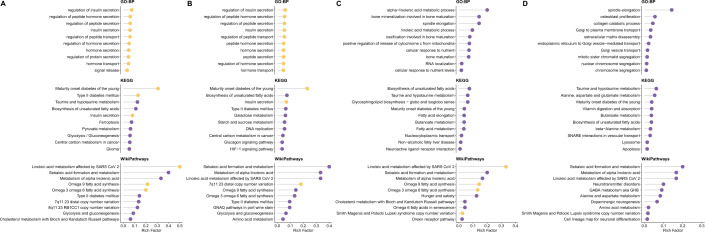
Comprehensive gene-set enrichment analysis of glycemic traits from meta-GWAS and TWAS. The top 10 enriched results from three databases were displayed, including GO: Biological Process, KEGG Pathway, and WikiPathways. **A** Enriched pathways for HbA1c-associated genes bases on meta-GWAS data; **B** Enriched pathways for fasting glucose-associated genes bases on meta-GWAS data; **C** Enriched pathways for HbA1c-associated genes bases on TWAS data; **D** Enriched pathways for fasting glucose-associated genes bases on TWAS data. Pathways with a false discovery rate (fdr) below 0.05 are highlighted in yellow, while others are shown in purple. The Rich factor is the ratio of the number of input genes annotated in a pathway to the total number of genes annotated in the same pathway

To gain deeper insights into the biological signatures, we conducted pathway enrichment analysis on the two glycemic traits. Using data from HbA1c meta-GWAS, 186 significant genes were identified. HbA1c-associated genes highlighted biological pathways that are involved in peptide and hormone secretion, specifically insulin secretion. This aligns with insulin's role as a peptide hormone in regulating blood sugar (Figure 4A, Additional file 2: Table S14). The enriched KEGG pathways underscore the importance of these genes in metabolic diseases like 'maturity onset diabetes of the young' (MODY) and ‘type II diabetes mellitus’, especially due to their connection with ‘insulin secretion’, linking them directly to the crucial process of glucose regulation. Moreover, we observed a notable connection between HbA1c levels and pathways related to linoleic acid metabolism and fatty acid synthesis. These findings suggest that disruptions in fatty acid metabolism, which is vital for cellular energy balance and insulin sensitivity, could indirectly influence glucose metabolism.

The meta-GWAS for FG identified 148 significant genes. The enriched biological processes in these genes mirror those associated with HbA1c, particularly highlighting insulin secretion as a key process (Figure 4B, Additional file 2: Table S15). Furthermore, the KEGG pathway analysis reveals a link between FG levels, insulin secretion, and the disease MODY. An interesting finding is the enrichment in the ‘7q11.23 distal copy number variation’ pathway, as identified by WikiPathways. While 7q11.23 CNV is typically linked to developmental disorders, its direct association with metabolic diseases is less evident. This enrichment could be attributed to the presence of multiple genes in the 7q11.23 region.

In parallel, we performed a similar analysis using TWAS results for comparative insights. With fewer input genes, most pathways did not meet the significant threshold (FDR < 0.05), suggesting a different underlying mechanism from the genome-based analysis. For HbA1c, 56 significant genes identified through TWAS were primarily enriched in metabolic processes, bone development and maturation, and cellular responses and regulation (Figure 4C, Additional file 2: Table S16). The KEGG pathways emphasized the regulation of fatty acid metabolism and MODY. WikiPathways indicated shared functionalities with genes from the meta-GWAS. For FG, TWAS identified 40 significant genes enriched in pathways involving cell division and chromosome segregation, bone and extracellular matrix processes, and intracellular transport (Figure 4D, Additional file 2: Table S17). Similar to the meta-GWAS findings, MODY was a consistent result. However, the WikiPathways results for FG presented a unique profile compared to other gene sets.

### Genetic profiles of glycemic traits forecast type 2 diabetes risk and offer insights for drug repurposing

**Fig. 5:**
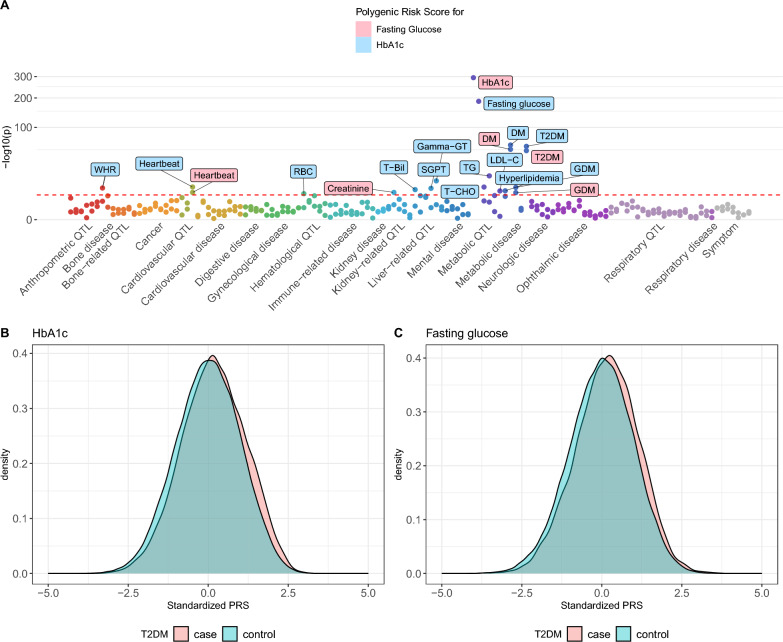
Polygenic risk score analyses. **A** Association between glycemic traits PRS and clinical traits from TWB. Red horizontal dashed lines indicate significance thresholds (−log10(1.9 × 10^–4^); **B** The risk of type 2 diabetes predicted by the PRS for HbA1c; **C** The risk of type 2 diabetes predicted by the PRS for fasting glucose

To explore how genetic factors linked to glycemic traits influence various phenotypes, we conducted association analyses between PRS for HbA1c and FG and 129 binary and quantitative phenotypes (Additional file 2: Table S18). The PRSs were calculated using significant variants from the meta-analysis of GWAS. We excluded any associations where the PRS originated from the same glycemic traits under study. Specifically, the association between HbA1c and HbA1c-derived PRS, as well as FG and FG-derived PRS were excluded to avoid overfitting. The results revealed significant associations between the glycemic trait PRS and several metabolic traits, including different forms of diabetes and blood lipid levels. Additionally, we discovered associations with unexpected traits, such as waist-hip ratio (WHR), heartbeat speed, red blood cell count (RBC), and phenotypes related to kidney and liver function (Fig. 5A). Furthermore, to assess the potential of PRSs as predictors of type 2 diabetes risk in the general population, we compared PRS values between individuals with and without type 2 diabetes from the TWB. Our analysis revealed that individuals diagnosed with type 2 diabetes exhibited higher PRS compared to those without the disease (Fig. 5B, C). This difference reached statistical significance for both HbA1c- and FG-based PRS (p-value < 2.2 × 10^–16^), underscoring the predictive value of these genetic markers for type 2 diabetes risk. We further investigated the development of type 2 diabetes over time by incorporating follow-up data from the TWB cohort. This analysis included 45,158 individuals with follow-up records. We discovered that 4.01% (168 out of 4185) of individuals with the top 10% of the PRS for HbA1c developed type 2 diabetes between their recruitment and the follow-up period. In contrast, only 2.61% (113 out of 4329) of individuals with the bottom 10% PRS for HbA1c developed type 2 diabetes later. Similarly, results from the PRS for FG revealed that 3.89% (164 out of 4217) of individuals in the top 10% group developed type 2 diabetes later on compared to only 2.6% (113 out of 4345) in the bottom 10% group. The mean follow-up period was 4.46 years.

Moreover, we integrated gene expression predictions from the TWAS into the iLINCs platform. By analyzing gene expression differences between diseased states and responses to specific treatments, we identified drugs capable of reversing disease effects. Specifically, we identified 118 drugs with the potential to modulate HbA1c levels (Additional file 2: Table S19) and 250 drugs that could potentially regulate FG (Additional file 2: Table S20), with 38 drugs common to both traits. To narrow down the candidates for treating type 2 diabetes, we excluded toxic compounds and focused on the remaining 29 therapeutic agents. Of these, chlorpropamide is already approved for type 2 diabetes treatment. A comprehensive literature review was performed to evaluate the treatment effects of these agents. This review led us to propose eight candidate drugs with potential benefits for serum glucose regulation (Table 2), including three used in oncology, two in immunology, and one each in analgesia, endocrinology, and psychiatry.

**Table 2:** Drug repurposing candidates for blood glucose regulation

Therapeutic area	Drug	Approved indication	References
Analgesia	Indomethacin	Pain	[17, 18]
Endocrinology	Estriol	Menopause	[19, 20]
Immunology	Cyclosporin A	Organ transplantation	[21]
Immunology	Leflunomide	Rheumatoid arthritis	[22]
Oncology	Erlotinib	NSCLC	[23, 24]
Oncology	Imatinib	Chronic myelogenous leukemia	[25–28]
Oncology	Tretinoin	Acute promyelocytic leukemia	[29]
Psychiatry	Sertraline	Major depressive disorder	[30]

## Discussion

This study constitutes the most comprehensive analysis of Taiwanese glycemic trait genetics, with a total sample size of 180,863. The single-ancestry meta-analysis identified 2148 shared risk loci between HbA1c and FG, all of which show consistent effect directions. This alignment not only suggests a potential biological interconnection between the regulation of HbA1c and FG but also underscores the complexity of their genetic determinants. Notably, rs113373052 (17q25.3) within the *FN3K* gene emerged as the top signal for HbA1c levels. This variant has been reported in multiple studies across diverse populations, including European, East Asian, Korean, Japanese, and Taiwanese populations [3, 31, 32]. Additionally, meta-GWAS for FG replicated the well-known variant rs10830963 (11q14.3) on *MTNR1B*, which has been reported in over 16 articles across Eastern and Western populations (Additional file 2: Table S10). The top signal for FG, rs1402837, exerted a correlated influence on HbA1c. A previous GWAS using data from the TWB identified several genetic variants associated with glycemic traits [3], and we successfully replicated all but one of the identified variants for both HbA1c and FG, respectively. Besides replicating previous findings, a total of 25 genes were highlighted to be newly associated with both HbA1c and FG.

At the transcriptome level, TWAS for HbA1c replicated 15.6% (29/186) of the significant findings from the meta-GWAS. Meanwhile, the TWAS for FG corresponded to 14.9% (22/148) of the significant findings in the meta-GWAS. The overlapping genes between GWAS and TWAS indicate that certain genetic risk variants influence gene expression, thereby affecting glycemic trait levels. Remarkably, the influence of six genes was corroborated at both genomic and transcriptomic levels. Among these, *YKT6*, *GAD2*, *FADS1*, and *FOXA2* were previously recognized for their impact on HbA1c and FG levels, while *MAEA* and *PRC1* have emerged as new discoveries from our analysis. *MAEA* is involved in erythroblast attachment to macrophages, and rs6815464 G > C was associated with type 2 diabetes in an East Asian GWAS meta-analysis [33]. Moreover, overexpression of *MAEA* in mouse hepatocytes has been shown to reduce hepatic gluconeogenesis, highlighting its potential as a therapeutic target for type 2 diabetes treatment [34]. *PRC1*, crucial for cytokinesis, has been identified as a susceptibility gene for type 2 diabetes [35, 36]. Pathway enrichment analysis revealed that risk genes associated with glycemic traits are involved in insulin secretion and diabetes. These findings suggest that genes influencing glycemic levels could impact the development or progression of specific types of diabetes, likely through mechanisms that affect insulin production or glucose metabolism.

Furthermore, the PRS analysis revealed that diabetes, blood lipids, and liver-related traits share genetic risk factors with HbA1c. The most significant associations were found with diabetes, followed by lipid profiles which are known to correlate with HbA1c levels [37]. Among liver-related traits, only total bilirubin was negatively associated with the PRS for HbA1c, with multiple studies confirming its independent negative correlation with HbA1c in individuals with and without type 2 diabetes [38, 39]. Additionally, serum creatinine levels were linked to the PRS for FG, with research by Yoshida et al. suggesting that low serum creatinine might indicate the onset of impaired FG due to changes in muscle volume from exercise habits [40]. Moreover, our results indicate that individuals in the highest PRS decile are more likely to develop type 2 diabetes over time compared to those in the lowest decile. This underscores the critical role of glucose regulation in the progression of this disease and highlights the potential of PRS as a tool for identifying genetically high-risk subpopulations. While PRS may not serve as a precise diagnostic tool for individual disease prediction, it holds promise for targeted prevention or monitoring strategies to mitigate diabetes risk at a population level.

Through drug repurposing, we identified eight drugs with clinical evidence supporting their effect on reducing blood glucose levels: indomethacin, estriol, cyclosporin A, leflunomide, erlotinib, imatinib, tretinoin, and sertraline. Indomethacin, a prostaglandin synthetase inhibitor, has shown variable effects on glucose levels; while one study suggested that it might impair glucagon function and lower blood glucose levels [18], others have found it could decrease insulin secretion and increase glucose production [41, 42]. Estrogens, including estriol, are thought to protect against diabetes by enhancing insulin secretion and regulating glucose homeostasis [43]. Cyclosporin A interacts with repaglinide to potentiate its hypoglycemic effect [21]. Leflunomide has been shown to inhibit p70 S6 kinase (S6K1), potentially sensitizing insulin receptors [22]. Tyrosine kinase inhibitors, including erlotinib and imatinib, are recognized for their hypoglycemic effects, which include preserving β-cell function and enhancing insulin sensitivity and secretion [44, 45]. Tretinoin significantly reduced FG and insulin levels in mice [29]. Sertraline, a selective serotonin reuptake inhibitor, enhances hypoglycemia [30] and is often prescribed to diabetic patients with depression, benefiting both conditions. Our findings could also help avoid hypoglycemia by considering the use of these candidate drugs in the treatment of type 2 diabetes. Many patients with type 2 diabetes often face multiple comorbidities, such as cardiovascular disease, hypertension, and kidney disorders, which necessitate the use of multiple medications. This polypharmacy increases the likelihood of complex drug-drug interactions, complicating treatment and potentially leading to adverse effects. Repurposing existing drugs for type 2 diabetes treatment offers a valuable approach in clinical practice by utilizing medications with well-understood safety profiles and interaction risks. This strategy could help reduce the challenges associated with polypharmacy and provide safer, more effective therapeutic options for type 2 diabetes patients with comorbid conditions [46]. However, some medications may not be suitable for repurposing in type 2 diabetes management due to immunosuppressive effects or toxicity. We plan to investigate these drugs' glucose-lowering effects using real-world data in future studies.

The study encountered several limitations. First, the TWB cohort lacked prescription data, preventing us from accounting for blood sugar-lowering medication use and its effects on HbA1c and FG levels. Secondly, a small number of variants were not assessed across both cohorts due to differences in genotyping arrays. Third, a potential limitation of our TWAS is that the weights are derived from likely disease-free European populations, which may affect the generalizability of our findings to populations with different genetic backgrounds and disease statuses. Variants contributing to gene expression in Europeans may not accurately represent the same eQTLs in Asians due to differences in allele frequencies and linkage disequilibrium patterns. Even when the same eQTLs are present in both populations, their regulatory effects on gene expression may vary. Rare or population-specific variants unique to Asians may be missed. To address this, future eQTL studies incorporating Asian populations and disease-relevant cohorts would be necessary to validate our findings. Moreover, the heritability of type 2 diabetes has been reported to range from 25% to 80% [47]. Although our study demonstrated the potential of PRS to predict type 2 diabetes risk, the influence of environmental factors cannot be overlooked. To assess these impacts on blood glucose regulation, future research will need to include data on participants' lifestyles and dietary habits. Lastly, as in vitro and in vivo studies were not conducted in this study, the lack of functional validation remains a limitation.

## Conclusions

In conclusion, this study uniquely integrates data from both community and hospital settings to capture a comprehensive spectrum of glucose control levels, ensuring a representation that closely reflects the general population in Taiwan. By incorporating diverse methodologies, encompassing genetics, transcriptomics, biological pathway analyses, polygenic risk scores, and drug repurposing for type 2 diabetes, our research provides a thorough understanding of the genetics and biological mechanisms underlying glucose regulation in Taiwanese individuals. Additionally, our identification of eight promising drug candidates for repurposing paves the way for future clinical validation, potentially leading to new therapeutic options for managing type 2 diabetes. Through this comprehensive approach, our study sheds light on the unique genetic landscape of glucose regulation in Taiwan, providing valuable insights that could guide future treatment strategies for type 2 diabetes.

## Supplementary Information

Additional file 1.

Additional file 2.

## Data Availability

The datasets used and/or analysed during the current study are available from the corresponding author on reasonable request.

## Abbreviations

HbA1c: Hemoglobin A1c
FG: Fasting glucose
PRS: Polygenic risk score
TWB: Taiwan Biobank
TCVGH: Taichung veterans general hospital
MAF: Minor allele frequency
HWE: Hardy–Weinberg equilibrium
GWAS: Genome-wide association study
TWAS: Transcriptome-wide association study
LD: Linkage disequilibrium
GOBP: Gene ontology biological process
KEGG: Kyoto encyclopedia of genes and genomes
MODY: Maturity onset diabetes of the young
