# Supplementary material for: Exploring the genomic and transcriptomic profiles of glycemic traits and drug repurposing
Source: J Biomed Sci. 2025 May 21;32:50. doi: 10.1186/s12929-025-01137-7 (PMC12096723; doi:10.1186/s12929-025-01137-7)
Supplement: Supplementary file 1 — Additional file 1. [file 12929_2025_1137_MOESM1_ESM.docx]

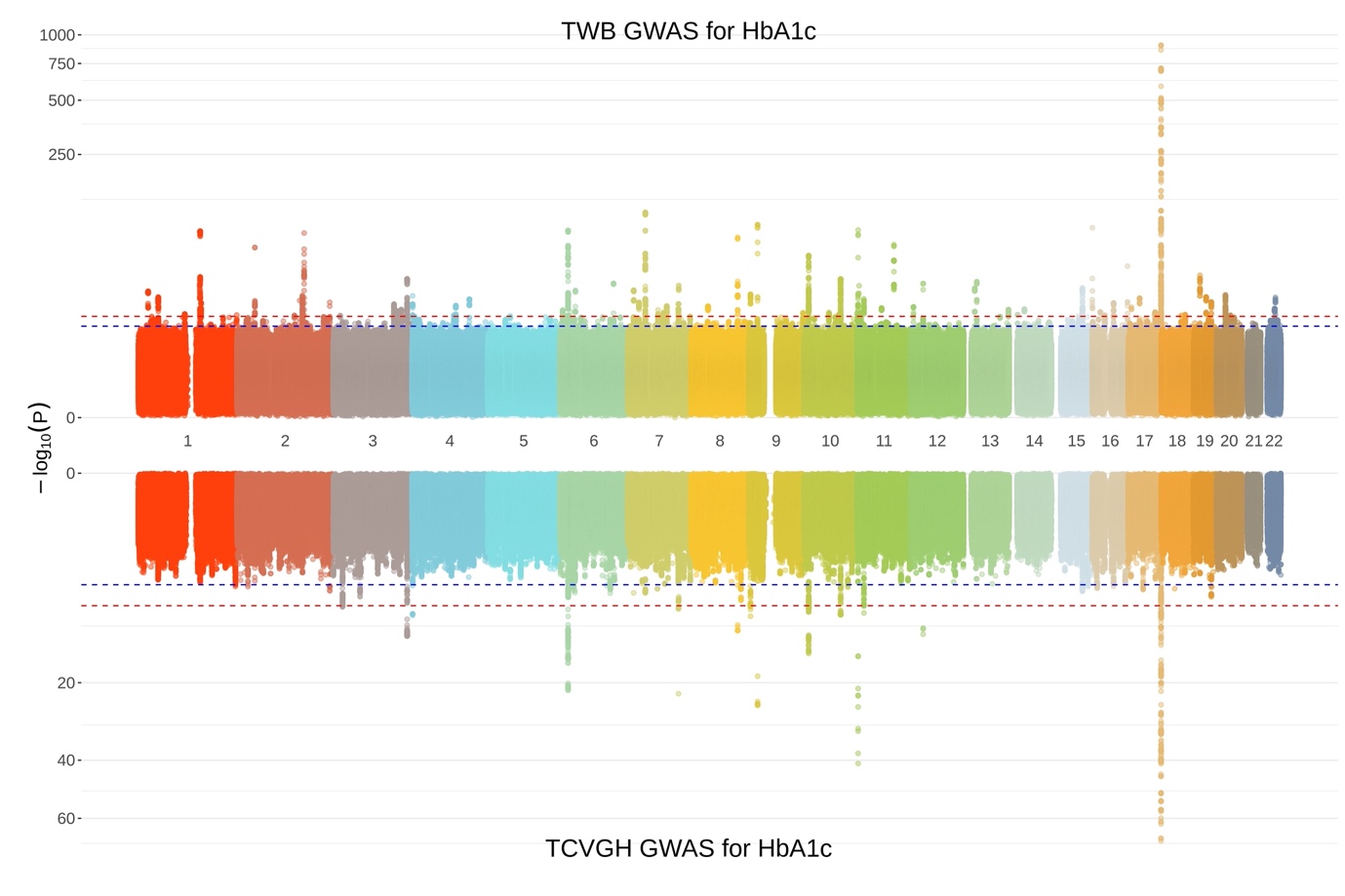


**Figure S1**. **Mirror Manhattan plot for HBA1C in the TWB (top) and TCVGH (bottom) cohorts.** Red horizontal dashed lines indicate the significance threshold (−log10(5×10^-8^). Blue horizontal dashed lines indicate suggestive significance threshold (−log10(1×10^-5^).

**
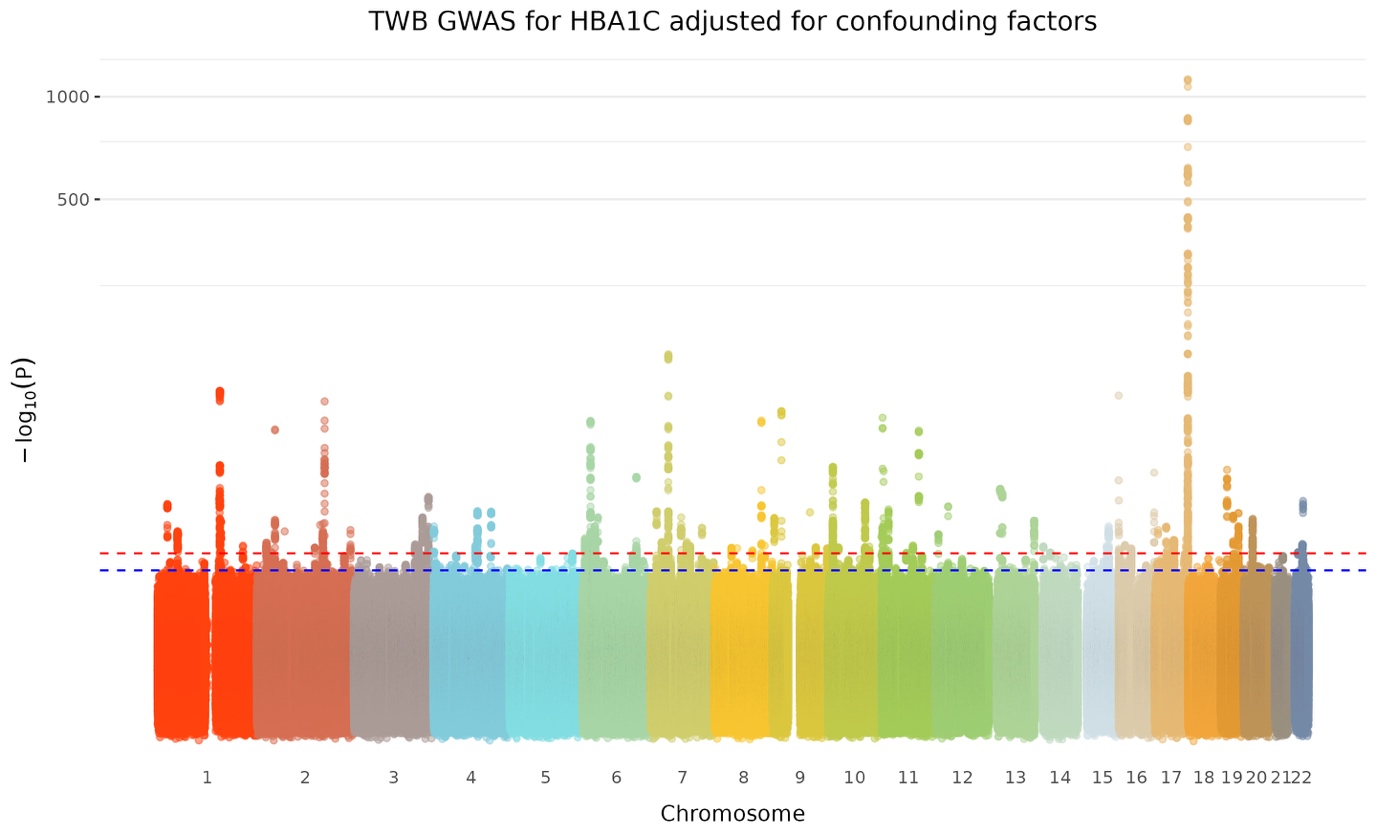
Figure S2**. **Manhattan plot for HBA1C in the TWB cohort adjusting for additional confounding factors.** Red horizontal dashed lines indicate the significance threshold (−log10(5×10^-8^). Blue horizontal dashed lines indicate suggestive significance threshold (−log10(1×10^-5^).


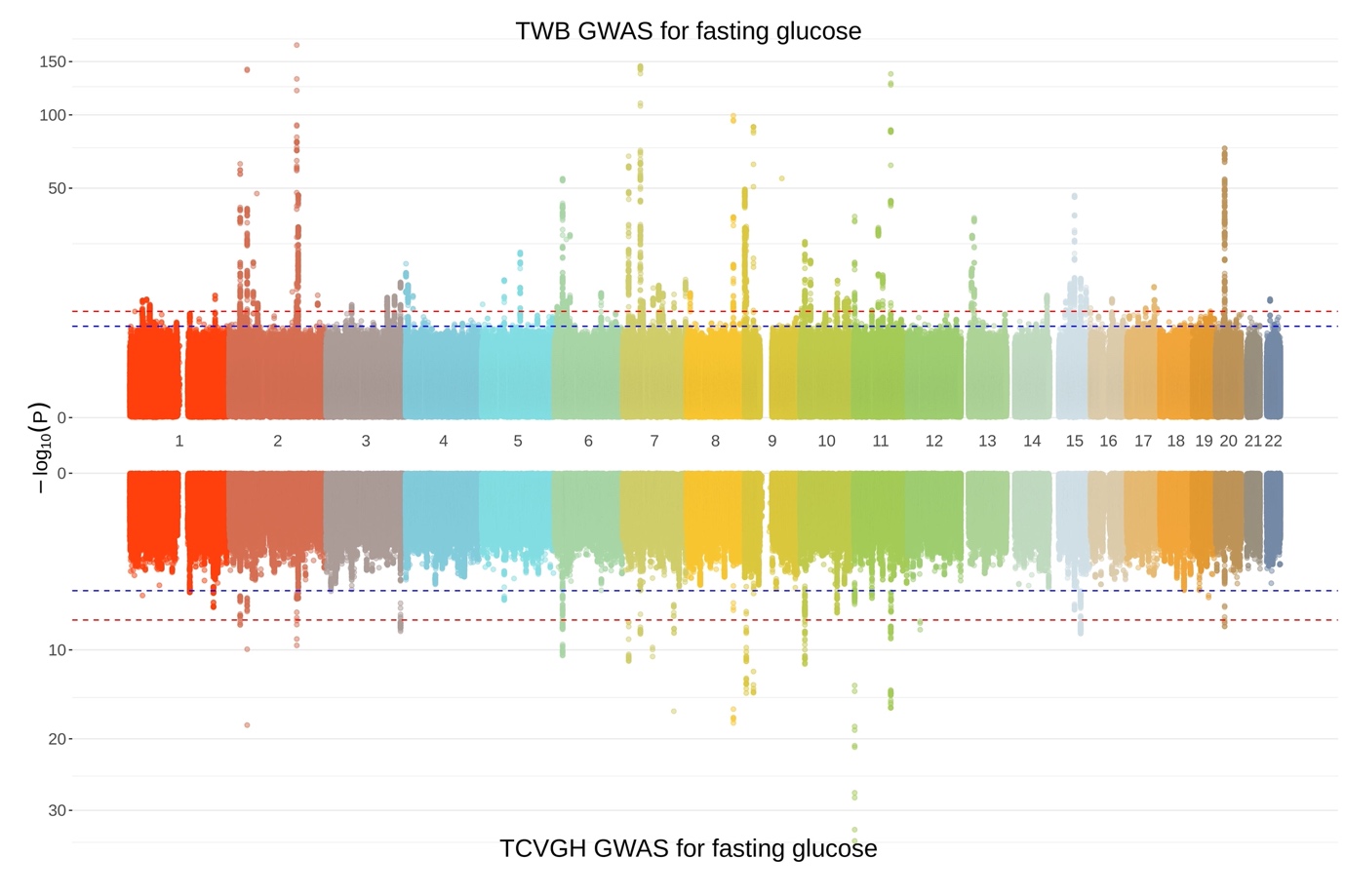
**Figure S3. Mirror Manhattan plot for fasting glucose in the TWB (top) and TCVGH (bottom) cohorts.** Red horizontal dashed lines indicate the significance threshold (−log10(5×10^-8^). Blue horizontal dashed lines indicate suggestive significance threshold (−log10(1×10^-5^).

**
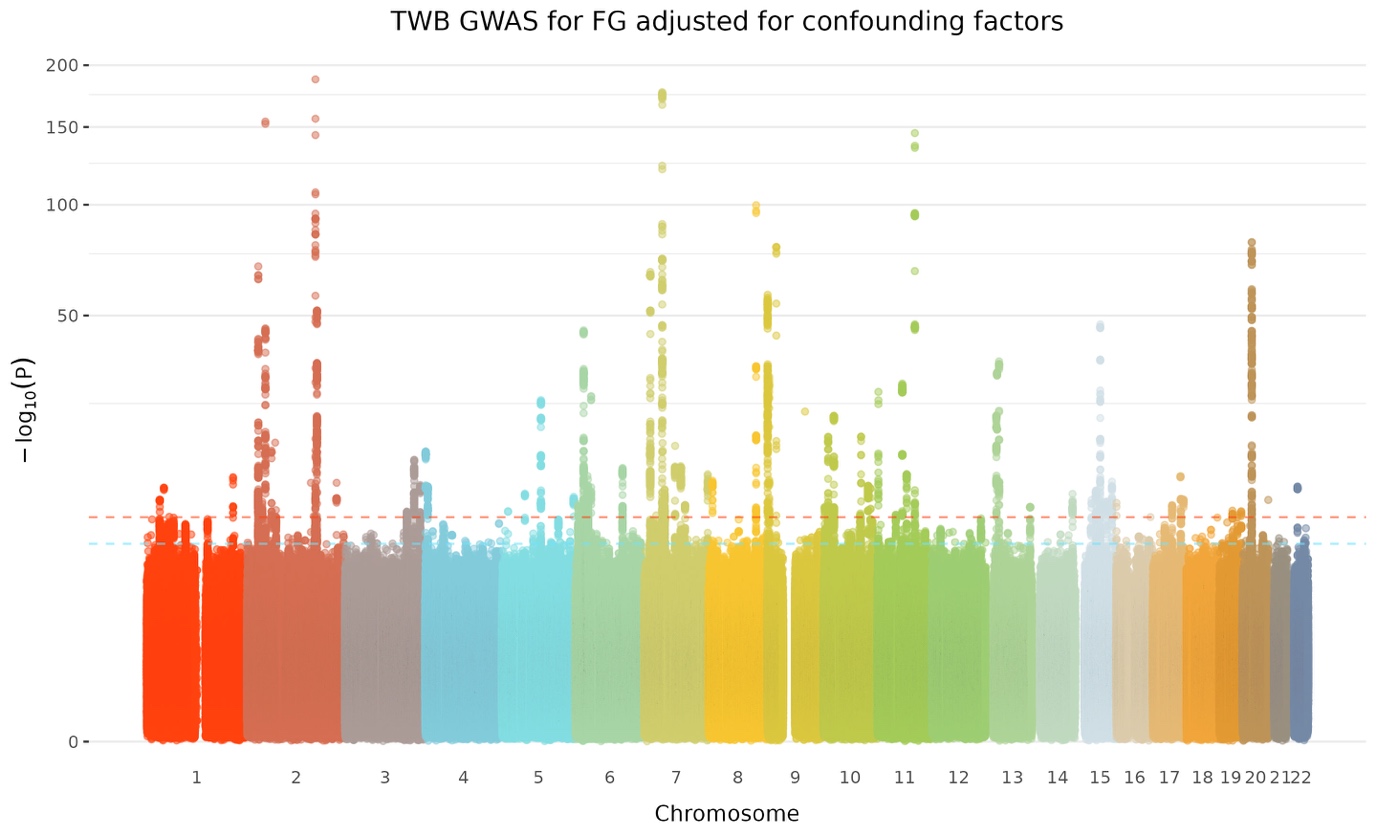
Figure S4**. **Manhattan plot for fasting glucose in the TWB cohort adjusting for additional confounding factors.** Red horizontal dashed lines indicate the significance threshold (−log10(5×10^-8^). Blue horizontal dashed lines indicate suggestive significance threshold (−log10(1×10^-5^).
